# The Dispensaries of Assam

**Published:** 1900-04-21

**Authors:** 


### THE DISPENSARIES OF ASSAM.

The dispensaries of the Province of Assam have now reached a total of over 100, of which 13 are first-class, 87 second-class, and one third-class; and seven new centres of relief were added during 1897. The classification of these dispensaries is not made according to size and importance, but first-class institutions are those which, except for some few private subscriptions, are entirely supported by funds derived from Government. The second-class are chiefly supported by local funds, though in 46 instances the hospital assistant is paid by Government, and grants of money are also occasionally made for the purchase of European medicines. The increase in the total number of patients treated in the 108 dispensaries during the 12 months was nearly 1,000, a very satisfactory result considering that 1898 was an unusually healthy year, and one which demonstrates that, however slow the growth may be, yet the public appreciation of the dispensaries is gradually assuming larger proportions. Of the in-patients, who numbered nearly 7,000, 4,000 were restored to health, 500 were relieved, and over 1,000 were discharged "otherwise," which means in the majority of cases that the patients thought they were not deriving sufficient benefit from residence in hospital, or not getting cured as rapidly as they wished, and therefore they absconded. Finally, no less than 1,121, or an average of 16·07, died in the wards. This, of course, is a fearfully high rate of mortality, although it was less than any previous period for nine years with the exception of 1896. In 1897 the percentage of deaths reached 20·94. In one particular the authorities are able to report a marked improvement. The number of people who sent their friends for medicine shows a marked decrease, and there is a corresponding increase in the number of those who personally applied for relief. This is a much more satisfactory state of affairs, and shows that the efforts to discourage applications for second-hand medical advice is bearing fruit. Comparatively, there was little cholera, but it is distinctly disagreeable to note that the number of victims to this disease is rising so steadily. It is possible that this may be due to better registration, but medical opinion rather inclines to the belief that cholera in Assam has become endemic, and that, till the rudiments of sanitation can be introduced into the villages, and the villagers be induced to eat, drink, die, and be disposed of in a cleanly manner, its presence will always be felt. Hitherto cases of kalá ázár have been classed with malarial fevers, but during the past year the total of cases treated has been kept in a separate register, by which it appears that during 1898 5,572 patients suffering from what was at one time called "anæmia of coolies" were treated in the dispensaries of Assam. This number is believed to be three or four thousand less than in the previous year, which gives ground for hope that the disease is on the wane, and that in the future it may gradually die out. The present system in vogue at many of the dispensaries with regard to severe cases of bowel complaint leaves much to be desired. There is an outside shed to which the name of "Moribund Ward" is attached, and to this most really serious cases seem to be relegated. That it is advisable for many reasons to separate those about to die from the rest of the inmates of a hospital ward is admitted, and severe cases of many diseases are better isolated as far as possible, but that is a very different thing from putting all acute cases of intestinal disease, some of which, though possibly incurable, may linger for days or weeks, into a hovel. At one dispensary the Sanitary Commissioner found a beautiful building, furnished with spring beds, clean linen, clock on the wall, flowers on the tables, &c., with room for 24 patients. Only six beds were occupied, and none of the six men were really very ill, while in a horrid little hut outside, fitly called the "Moribund Ward," were nine cases of serious illness—not one of which was, however, by any means hopeless—crammed together in less than half the space they should have occupied. As the Commissioner pertinently adds, "I like to see clean and well-kept wards, but I would rather have them made use of, even if not so spick and span, than kept for show, while the real mass of misery which the institution is meant to alleviate is kept in the background."

